# Supplementary material for: Self-care practice and its barriers among diabetes patients in North East Ethiopia: A facility-based cross-sectional study
Source: PLOS Glob Public Health. 2024 Feb 15;4(2):e0002036. doi: 10.1371/journal.pgph.0002036 (PMC10868755; doi:10.1371/journal.pgph.0002036)
Supplement: S1 Annex — (PDF) [file pgph.0002036.s001.pdf]

S1 Annex: Questionnaire

Annex-I:

English Version Consent form

Part-I Subject Information Sheet

Principal investigator: Adisu Asefa

Organization: Debre Berhan University

My name is Adisu Asefa student of Debre Berhan University Department of public health. I am doing a research on "Barriers to Life-Style and Dietary Pattern Intervention Practice among Type-2 Diabetes Patients Visiting Debre Berhan Town Public Health Institutions, North Shewa Zone, Amhara National Regional State, Ethiopia." I received permission letter from Debre Berhan University Department of public health and Public health Institutions.

You are selected by systematic random sampling method to participate in this study because you are currently attending diabetic follow up. Your participation is purely based on your willingness. You have the right to choose not to take part in this study. If you choose to take part, you have the right to stop at any time. If you are willing to participate or refuse or decide to withdraw later, you will not be subjected to any ill-treatment.

If you agree to participate in the study, you will be asked to answer some questions about yourself, your life-style and dietary pattern practice. The interview with you will take about 30 minutes.

The study will investigate the magnitude and barriers to life-style and dietary pattern intervention which might help to design strategies to foster adherence and compliances to the interventions. It can also provide base line data for policy makers and other researchers for further improvements in diabetic life-style modifications. The information that you provide will be kept confidential by using only code numbers. Do not give your name. No one will have access to the non-coded data except the principal investigator and the data will not be used for purposes other than the study. Your willingness and active participation is very important for the success of this study.

Address: Adisu Asefa Tel. +251912464355 Email: sadamasefadb@gmail.com

Questionnaires ID__

Part-II: Informed Consent Form

Based on the understanding of the information and I have given information, are you willing to participate in this study?

A) Yes

B) No

(1) If yes, I will continue

2) If no I will skip to next participant after writing the reasons of refusal.

Respondent

Signature ______________________Date__________________

Interviewer

Name____________________________________ Signature________________

Questionnaires number_____________________

Date of interview___________________ Starting time_________ Completed________

Result of interview A) Completed

B) Not completed

C) Partially completed

D) Refused

Checked by Supervisor: Name ________________________Signature_____________

Address: Adisu Asefa Tel. +251912464355

Email: sadamasefadb@gmail.com

Instruction: circle all the possible answers of the respondent from the choice provided.

English Version Questionnaire

Questionnaire ID __________

Part-I: Socio-demographic characteristics of type 2 diabetes patients visiting Debreberhan Town Public Health Institutions

No	Variables	Options
101.	Sex	1. Male<br>2. Female
102.	Age	
103.	Educational level	1. Illiterate<br>2. Read and Write<br>3. Primary School<br>4. Secondary School<br>5. College/ University
104.	Marital Status	1. Single<br>2. Married<br>3. Widowed<br>4. Divorced
105.	Religion	1. Orthodox<br>2. Muslim<br>3. Protestant<br>4. Catholic<br>5. Others
106.	Occupation	1. Physical labor<br>2. Teacher<br>3. Health professional<br>4. Accountant and Business man<br>5. Engineer<br>6. Private work<br>7. House wife<br>8. Driver<br>9. Others
107.	Monthly Income	1. Very Low<br>2. Low<br>3. Average<br>4. Above average<br>5. High

Part-II: d of type 2 diabetes patients visiting Debreberhan Town Governmental Health Institutions

No	Variables	Options
201.	Duration of the disease	1. Less than one year<br>2. One year to five years<br>3. Greater than five years
202.	Family history of diabetes	1. Yes<br>2. No
203.	Treatment intensity	1. Oral Hypoglycemic agent<br>2. Insulin therapy<br>3. Both
204.	Social support	1. Yes<br>2. No
205.	Currently do you have your own glucometer at home	1. Yes<br>2. No
206.	Diabetes complications	1. Yes<br>2. No
207.	Have you smoked a cigarette, even a puff, in the past SEVEN DAYS	1. Yes<br>2. No

Part-III: The Summary of diabetes self-care activities of type 2 diabetes patients visiting Debreberhan Town Governmental Health Institutions

No	Variables	Options (How many of the last 7 days)							
		**0**	**1**	**2**	**3**	**4**	**5**	**6**	**7**
301-304	**Diet**<br>301. How many of the last SEVEN DAYS have you followed a healthful eating plan								
	302. On average, over the past month, how many DAYS PER WEEK have you followed eating plan?								
	303. On how many of the last SEVEN DAYS did you eat five or more servings of fruits and vegetables?								
	304. On how many of the last SEVEN DAYS did you eat high fat foods such as red meat or full-fat dairy products?								
305-306	**Exercise**<br>305. On how many of the last SEVEN DAYS did you participate in at least 30 minutes of physical activity? (Total minutes of continuous activity, including walking).								
	306. On how many of the last SEVEN DAYS did you participate in a specific exercise session (such as swimming, walking, biking) other than what you do around the house or as part of your work?								

307-308	**Blood Sugar Testing**<br>307. On how many of the last SEVEN DAYS did you test your blood sugar?								
	308. On how many of the last SEVEN DAYS did you test your blood sugar the number of times recommended by your health care provider?								
309-310	**Foot Care**<br>309. On how many of the last SEVEN DAYS did you check your feet?<br>310. On how many of the last SEVEN DAYS did you inspect the inside of your shoes?								
311-312	**Smoking**<br>311. Have you smoked a cigarette even one puff during the past SEVEN DAYS?<br>312. If yes, how many cigarettes did you smoke on an average day?<br>Number of cigarettes: __	1. No<br>2. Yes							

Part-III: Additional Items for the Expanded Version of the Summary of Diabetes Self-Care Activities.

A. Self-Care Recommendations

No	Variables	Options
**401.**	Which of the following has your health care team (doctor, nurse, dietitian, or diabetes educator) advised you to do? Please check all that apply:	a. Follow a low-fat eating plan<br>b. Follow a complex carbohydrate diet<br>c. Reduce the number of calories you eat to lose weight<br>d. Eat lots of food high in dietary fiber<br>e. Eat lots (at least 5 servings per day) of fruits and vegetables<br>f. Eat very few sweets (for example: desserts, non-diet sodas, candy bars)<br>g. Other (specify):<br>h. I have not been given any advice about my diet by my health care team.
**402.**	Which of the following has your health care team (doctor, nurse, dietitian, or diabetes educator) advised you to do? Please check all that apply:	a. Get low level exercise (such as walking) on a daily basis.<br>b. Exercise continuously for a least 20 minutes at least 3 times a week.<br>c. Fit exercise into your daily routine (for example, take stairs instead of elevators, park a block away and walk, etc.)<br>d. Engage in a specific amount, type, duration and level of exercise.<br>e. Other (specify):<br>f. I have not been given any advice about exercise by my health care team.
**403.**	Which of the following has your health care team (doctor, nurse, dietitian, or diabetes educator) advised you to do? Please check all that apply:	a. Test your blood sugar using a drop of blood from your finger and a color chart.<br>b. Test your blood sugar using a machine to read the results.<br>c. Test your urine for sugar.<br>d. Other (specify):<br>e. I have not been given any advice either about testing my blood or urine sugar level by my health care team
**404.**	Which of the following medications for your diabetes has your doctor prescribed? Please check all that apply.	a. An insulin shot 1 or 2 times a day.<br>b. An insulin shot 3 or more times a day.<br>c. Diabetes pills to control my blood sugar level.<br>d. Other (specify):<br>e. I have not been prescribed either insulin or pills for my diabetes.

B. Self- care activities practice

No	Variables	Options (How many of the last 7 days)							
		0	1	2	3	4	5	6	7
501.	**Diet**<br>501. How many of the last SEVEN DAYS did you space carbohydrates evenly through the day?								
502-504	**Medication**<br>502. On how many of the last SEVEN DAYS, did you take your recommended diabetes medication?								
	**503.** On how many of the last SEVEN DAYS did you take your recommended insulin injections?								
	504. On how many of the last SEVEN DAYS did you take your recommended number of diabetes pills?								
505-507	**Foot Care**<br>**505.** On how many of the last SEVEN DAYS did you wash your feet?								
	**506.** On how many of the last SEVEN DAYS did you did you soak your feet?								
	507. On how many of the last SEVEN DAYS did you dry between your toes after washing?								
508-510	**Smoking**<br>**508.** At your last doctor's visit, did anyone ask about your smoking status?	1. No<br>2. Yes							
	**509.** If you smoke, at your last doctor's visit, did anyone counsel you about stopping smoking or offer to refer you to a stop-smoking program?	1. No<br>2. Yes<br>3. Do not smoke.							

	510. When did you last smoke a cigarette?	a. More than two years ago, or never smoked<br>b. One to two years ago<br>c. Four to twelve months ago<br>d. One to three months ago<br>e. Within the last month<br>f. Today

Part IV: Barriers for non-adherence to dietary and lifestyle intervention

No	Variables	Options
**601.**	Which of the following barriers are responsible for poor dietary adherence?<br>Please check all that apply	a. Eating out (in restaurant<br>b. Financial constraints<br>c. Poor self-discipline<br>d. Eating at another's home<br>e. Situation at home<br>f. Lack of information on a healthy diet<br>g. Others
**602.**	Which of the following barriers are responsible for poor exercise adherence? Please check all that apply	a. Weather condition (very cold winters and very hot summers)<br>b. Lack of exercise partner<br>c. Specific location<br>d. Criticism<br>e. Lack of information about the benefit of exercise and how it should be done<br>f. The notion that exercise exacerbated diabetes mellitus<br>g. Others

Amharic Version Consent form

ደብረ ብርሃን ዩኒቨርስቲ

ህብረተሰብ ጤና ሳይንስ

የተጠያቂው / መላሾች የመረጃ ቅፅ

እንደምን አደሩ / ዋሉ ፡፡ ስሜ -------------------------------- ይባላል:: የመጣሁት ከደብረ ብርሃን ዩኒቨርስቲ ህብረተሰብ ጤና ሳይንስ ሲሆን፡፡ ሁለተኛ ዲግሪዬን ለመመረቅ በደብረ ብርሃን ከተማ በሚገኙ የመንግስት ጤና ተቋማት ላይ የስኳር ህክምና ክትትል በሚያደርጉ ግለሰቦች ላይ ሲሆን ከደብረ ብርሃን ዩኒቨርስቲ እና ከመንግስት ጤና ተቋማት ፍቃድ አግኝቼ ምርምር ና ጥናት እየሰራሁ ነው፡፡

እርስዎ የተመረጡት በዚህ ጤና ተቋማት የስኳር ህክምና ክትትል በማድረጎ ነው፡፡በመሆኑም ሁለተኛ የስኳር አይነት ያላቸዉ ስኳር ህመምተኞች ይሳተፋሉ፡፡ የእርስዎ ተሳትፎ ሙለ በሙሉ የእርስዎ ፈቃደኝነት , ላይ የተመሰረተና በጥናቱ መሳተፍ ያለመሳተፍ መብት አልዎት፡፡ ለመሳተፍ ፈቃደኛ ከሆኑ በኋላም በፈለጉት ጊዜ ማቋረጥ ወይም ማቆም ይችላሉ፡፡ በጥናቱ ባለመሳተፍዎ የሚደርስቦት ምንም አይነት ችግር አይኖርም፡፡ በጥናቱ ለመሳተፍ ከተስማሙ ስለ አመጋገብዎ እና ስለ ህይወት ዘይቤዋ አተገባበር ላይ ያሉትን ልምድ እስከ 30 ደቂቃ ሊወስድ የሚችሉ የተወሰኑ ጥያቄዎች እንጠይቆታለን፡፡

ከጥናቱ ተገቢውን አመጋገብ ና የህይወት ዘይቤዋ አተገባበር እንዴት ማሻሻል እንዳለብዎ ይረዳሉ፡፡ ከዚህም በተጨማሪ የጥናቱ ውጤት የስኳር ህመምተኞች አመጋገብ ና የህይወት ዘይቤዋ ይበልጥ ለማሻሻል ለተመራማሪዎች በዚህ ዙሪያ ለሚሰሩ አካላት እንደ መነሻ ያገለግላል፡፡

ለማንኛውም አይነት ጥያቄ ፡፡ ሞባይል ቁጥር: +251912464355 ኢሜል፡ sadamasefadb@gmail.com

የስምምነት መጠየቂያ/ ማረጋገጫ ቅፅ

ከላይ በሰጠሁዎት መረጃ መሰረት በዚህ ጥናት መሳተፍ ፈቃደኛ ነዎት

1) አዎ

የመጠይቁ ቁጥር ______________________

የጤና ተቋሙ ስም _____________________

መጠይቁ የተካሄደበት ቀን

መጠይቁ የተጀመረበት ሰአት

መጠይቁ የተጠናቀቀበት ሰአት

ጠያቂ ስም_________________ ፊርማ _________________

ተቆጣጣሪ ስም_________________ ፊርማ ______________ቀን____

የቃለ መጠይቁ ዉጤት

1) ሙለ በሙለ የተሞላ

2) በከፊል የተሞላ

3) ምንም ያልተሞላ

(2) አይደለሁም ፍቃደኛ ካልሆኑ (ምክንያቱን ወደ ሚቀጥለዉ ተሳታፊ እለፍ)

ለማንኛውም አይነት ጥያቄ ። ሞባይል ቁጥር: +251912464355 ኢሜል፥ sadamasefadb@gmail.com

ትእዛዝ ፤ተሳታፊዎቹ የሚሰጡትን ማንኛዉንም መልስ ከተሰጡት አማራጮች ዉስጥ ለይተዉ አክብብ

8.1.4 Amharic Version Questionnaire form

የመጠይቁ ቁጥር ________________

ክፍል 1: ከዚህ በመቀጠል አንዳንድ ጥያቄዎች ልጠይቅዎ እወዳለዉ ምላሽዎትን ከማነበው ምርጫ ዉስጥ የትኛዉ እንደሆነ ይነግሩኛል

ቁጥር	ጥያቄ	ምላሽ
101.	ፆታ	1. ወንድ<br>2. ሴት
102.	ዕድሜ	
103.	የትምህርት ደረጃ	1. ያልተማረ<br>2. ማንበብ ና መፃፍ<br>3. የመጀመሪያ ደረጃ<br>4. ሁለተኛ ደረጃ<br>5. ኮሌጅ/ ዩኒቨርስቲ
104.	የጋብቻ ሁኔታ ?	1. ያላገባ<br>2. ያገባ<br>3. የሞተባት<br>4. የፈታ
105.	ሃይማኖት	1. ኦርቶዶክስ<br>2. ሙስሊም<br>3. ፕሮቴስታንት<br>4. ካቶሊክ<br>5. ሌላ
106.	የስራ ድርሻ	1. የቀን ሰራተኛ<br>2. መምህር<br>3. የጤና ባለሙያ<br>4. የቢዝነስ ስራ<br>5. መሃንዲስ<br>6. የግል ስራ<br>7. የቤት እመቤት<br>8. ሹፌር<br>9. ሌላ
107.	ወርሃዊ ገቢዎ<br>ምን ያህል ነዉ?	

ክፍል ሁለት፡ ከዚህ በመቀጠል ስለ ጤናዎት ሁኔታ አንዳንድ ጥያቄዎች እጠይቅዎታለዉ ምላሽዎትን ከማነብሎት ምርጫ ዉስጥ ይገልጹልኛል

ተ.ቁ	ጥያቄ	ምላሽ
201.	የስኳር ህመም ከጀመሮት ምን ያህል<br><br>ጊዜ ሆኖት?	1. ከ 1 ዓመት በታች<br>2. ከ 1 ዓመት እስከ 5 ዓመት<br>3. ከ 5 ዓመት በላይ
202.	በዘር (በቤተሰብዎ) የስኳር ህመም<br><br>አለ?	1. አዋ<br>2. የለም
203.	የስኳርዎን መጠን ለማስተካከል<br><br>በየትኛዉ መንገድ ይጠቀማሉ?	1. በአፍ የሚዋጥ<br>2. በኢንሱሊን<br>3. ሁለቱንም
204.	የቤተሰብ/የማህበረሰብ ድጋፍ ያገኛሉ	1. አዋ<br>2. የለም
205.	በአሁኑ ጊዜ የራስዋ የስኳር መለኪያ መሳሪያ በቤትዋ ይጠቀማሉ	1. አዋ<br>2. የለም
206.	የስኳር በሽታ ተያያዥ ችግሮች አሎት	1. አዋ<br>2. የለም
207.	ሲጋራ ያጨሳሉ	1. አዋ<br>2. የለም

ክፍል-3፡ የስኳር ታማሚዎች የአመጋገብ ና የህይወት ዘይቤዎች ተግባራትን በተመለከተ

ተ.ቁ	ጥያቄ	ምላሽ (ባለፈው 7 ቀን ውስጥ ምን ያህል ቀን)							
		0	1	2	3	4	5	6	7
301-304	አመጋገብ<br>301. ጤናማ የ አመጋገብ ዕቅድ ተከትለዋል								
	302. በአማካይ፤ ባለፈው ወር ውስጥ በሳምንት ምን ያህል ቀን ጤናማ የ አመጋገብ ዕቅድ ተከትለዋል								
	303. በቀን 5 ና ከዚያ በላይ ፍራፍሬና አትክልት ያለበት ምግብ በገበታዋ ተጠቅመዋል								
	304. ከባድ ቅባት አዘል ምግቦች ለምሳሌ ቀይ ስጋ ወይም የስጋና የወተት ተዋህጾ ተመግበዋል ተመግበዋል								
305-306	የአካል ብቃት እንቅስቃሴ<br>305. የአካል ብቃት እንቅስቃሴ ቢያንስ ለ 30 ደቂቃ ሰርተዋል(የተለያዩ የስራ እንቅስቃሴዎች፣ እርምጃ)								
	306. የተወሰኑ የአካል ብቃት እንቅስቃሴ ክፍለ ጊዜዎች ላይ ተሳትፈዋል (ዋና፣እርምጃ፣ሳይክል መንዳት) ቤትዋ አቅራቢያ ወይም ከሰራዋ ውጪ በሆነ መንገድ)								

307-308	የደም ስኳር መጠንን መለካት የተመለከተ<br><br>307. የደም ስኳር መጠንዋን ተለክተዋል								
	308. ጤና ባለሙያዎች ያዘዙትን ቁጥር ያህል ተለክተዋል								
309-310	የእግር ጤናዋትን በተመለከተ<br>309. የእግሮትን ጤንነት ተመልክተዋል								
	310. የእግሮትን የውስጥ ክፍል ጤንነት ተመልክተዋል								
311-312	ማጨስን በተመለከተ<br>311. ሲጋራ አጭሰዋል፤ 1 ፓኬት ቢሆንም	1. አዋ<br>2. የለም							
	312. አዋ ከሆነ መልሰዋ በአማካይ በቀን ምን ያህል አጭሰዋል								

ክፍል-3፡ ተጨማሪ የስኳር ታማሚዎች የአመጋገብ ና የህይወት ዘይቤዎች ተግባራትን በተመለከተ

በጤና ባለሙያዎች የታዘዙ ራስን የመጠበቅ ተግባራት

ተ.ቁ	ጥያቄ	ምላሽ
401.	ከሚከተሉት ተግባራት ውስጥ የጤና ባለሙያ (ዶክተር፣ነርስ፤ስነምግብ፣ የስኳር ትምህርት ባለሙያ) ምክር ሰጥዋታል፡፡<br>እባክዎት ዝርዝሮቹን ተመልከቱና የሚመለከቶትን ይግለፁ	1. ቅባት መጠናቸው ዝቅተኛ የሆነ ምግብ መመገብን መከተል<br>2. ከባድ ሀይል ሰጪ የሆነ ምግብ መመገብን መከተል<br>3. ክብደት ለመቀነስ የሚመገቡትን የካሎሪ መጠን መቀነስ<br>4. ከፍተኛ የፊይበር መጠን ያላቸውን ምግቦችን በብዛት መመገብ<br>5. 5 ና ከዚያ በላይ ቀን ፍራፍሬና አትክልት ያለበት ምግቦችን በብዛት ተመገበዋል<br>6. በጣም ጣፋጭ የሆኑ ምግቦች (ኬክና ብስኩቶች፣ከረሜላ)<br>7. ሌላ<br>8. ምንም አይነት ምክር አልተቀበልኩም
402.	ከሚከተሉት ተግባራት ውስጥ የጤና ባለሙያ (ዶክተር፣ነርስ፤ስነምግብ፣ የስኳር ትምህርት ባለሙያ) ምክር ሰጥዋታል፡፡<br>እባክዎት ዝርዝሮቹን ተመልከቱና የሚመለከቶትን ይግለፁ	1. ቀለል ያሉ እንቅስቃሴዎች ለምሳሌ እርምጃ በ የቀኑ መስራት<br>2. በተከታታይ ቢያንስ ለ 20 ደቂቃዎች በሳምንት 3 ቀናት የአካል ብቃት እንቅስቃሴ መስራት<br>3. በየቀኑ ህይወትዋ ላይ የአካል ብቃትዋን ማጎልበት (ለምሳሌ ሊፍት ከመጠቀም ደረጃ መጠቀም፣ መኪናዋትን ፓርክ አድርገው በ እግር መንቀሳቀስ)<br>4. በተወሰኑ አካላዊ እንቅስቃሴዎች ላይ መሳተፍ<br>5. ሌላ<br>6. ምንም አይነት ምክር አልተቀበልኩም
403.	ከሚከተሉት ተግባራት ውስጥ የጤና ባለሙያ (ዶክተር፣ነርስ፤ስነምግብ፣ የስኳር ትምህርት ባለሙያ) ምክር ሰጥዋታል፡፡<br>እባክዎት ዝርዝሮቹን ተመልከቱና የሚመለከቶትን ይግለፁ	1. የስኳር መጠኖን ከጣትዋ ላይ ደም በመውሰድ በከለር ቻርት መለካት<br>2. የስኳር መጠኖን ማሽን በመጠቀም ማንበብን<br>3. ስኳሮን ሽንት በመውሰድ መለካት<br>4. ሌላ<br>5. ምንም አይነት ምክር አልተቀበልኩም
404.	ለስኳር መጠኖን ለማስተካከል ከሚከተሉት መድሃኒቶች የትኞቹ በጤና ባለሙያዎች ታዘውሎታል<br>እባክዎት ዝርዝሮቹን ተመልከቱና የሚመለከቶትን ይግለፁ	1. ኢንሱሊን 1 ወይም 2 ሾት በ ቀን<br>2. ኢንሱሊን 3 ወይም ከዚያህ በላይ ሾት በቀን<br>3. .የስኳር መጠንዋን ለመቆጣጠር እንክብሎችን መውሰድ<br>4. ሌላ<br>5. ምንም አይነት ምክር አልተቀበልኩም

ራስን የመጠበቅ ተግባራት

ተ.ቁ	ጥያቄ	ምላሽ (ባለፈው 7 ቀን ውስጥ ምን ያህል ቀን)							
		0	1	2	3	4	5	6	7
501.	አመጋገብ<br>a. በቀን ውስጥ ሀይል ሰጪ ምግቦችን አራርቀው ይጠቀማሉ								
502-504	መድሀኒቶችን በተመለከተ<br>502. በጤና ባለሙያ የታዘዘሎትን መድሃኒት ወስደዋል								
	503. በጤና ባለሙያ የታዘዘሎትን ኢንሱሊን መርፌ ወስደዋል								
	504. በጤና ባለሙያ የታዘዘሎትን የመድሃኒት ብዛት ቁጥር ወስደዋል								
505-507	የእግር ጤናን በተመለከተ<br>505. እግሮትን ታጥበዋል								
	506. እግሮትን በሙቅ ውሃ ተዘፍዝፍዋል?								
	507. እግሮትን ከታጠቡ ቡዋላ የጣቶትን መካከል አድርቀዋል								
508-510	ማጨስን በተመለከተ<br>508. በባለፈው የህክምና ክትትሎ ማጨስን በተመለከተ ተጠይቀዋል	1. የለም<br>2. አዋ							
	509. በባለፈው የህክምና ክትትሎ አጭሰው ከሆነ፣ ማጨስዋን እንዲያቆሙ ተመክረዋል	1. የለም<br>2. አዋ<br>3. አላጨስቁም							
	510. ለመጨረሻ ጊዜ ያጨስከው መቼ ነው	1. ከሁለት ዓመት በፊት ወይም ፈጽሞ አላጨስኩም<br>2. ከ 1 ወይም 2 ዓመት በፊት<br>3. ከ 4-12 ወራት በፊት<br>4. ከ1-3 ወራት በፊት<br>5. ባለፈው ወር<br>6. ዛሬ							

ክፍል-4፡ የአመጋገብ ና የህይወት ዘይቤዎትን ተግባራዊ እንዳይሆኑ የሚያደርጉ ተግዳሮቶች በተመለከተ

ተ.ቁ	ጥያቄ	ምላሽ
601.	ከሚከተሉት ውስጥ የትኞቹ ተግዳሮቶች<br>የአመጋገብ ዘይቤዎት ተግባራዊ እንዳይሆኑ አድርገዋል<br>እባክዎት የሚመለከቶትን ከበቡ	1. ውጪ ተመግቦ መግባት(ሬስቶራንት፣)<br>2. የ ኢኮኖሚ አቅም ሁኔታ<br>3. ደካማ የራስ ባህሪ<br>4. ሌላ ቦታ መመገብ<br>5. በቤት ውስጥ ያለ ሁኔታ<br>6. ስለ ጤናማ አመጋገብ መረጃ ማጣት<br>7. ሌላ ካለ ይግለፁ
602.	ከሚከተሉት ውስጥ የትኞቹ ተግዳሮቶች<br>የአካል ብቃት ዘይቤዎት ተግባራዊ እንዳይሆኑ አድርገዋል<br>እባክዎት የሚመለከቶትን ከበቡ	1. የአየር ሁኔታ (በጣም ቀዝቃዛ ወይም በጣም ሞቃት መሆን)<br>2. አብሮ እንቅስቃሴ የሚያደርግ ግለሰብ ማጣት<br>3. የተወሰነ ቦታ ማጣት<br>4. ትችት<br>5. ስለ አካል ብቃት እንቅስቃሴ ና እንዴት መስራት እንዳለብኝ መረጃ ማጣት<br>6. እንቅስቃሴ የስኳር በሽታ ያባብሳል ብሎ ማሰብ<br>7. ሌላ ካለ ይግለፁ
