# Supplementary material for: Self-care practice and its barriers among diabetes patients in North East Ethiopia: A facility-based cross-sectional study
Source: PLOS Glob Public Health. 2024 Feb 15;4(2):e0002036. doi: 10.1371/journal.pgph.0002036 (PMC10868755; doi:10.1371/journal.pgph.0002036)
Supplement: Attachment — Submitted filename: Edited Response to Reviewers.docx [file pgph.0002036.s002.docx]

Date: 16 May 2023

**Dear the editor and reviewers of PLOS Global Public Health,**

I would like to appreciate all of you for considering my manuscript for your journal. I highly appreciate your patience to give me adequate time to revise the manuscript based on the points raised by the editor and reviewers.

I would like to thank the editor and reviewers for giving me a nice comments and suggestions. I appreciate the points raised during the review process. After saying this, I would like to address the points raised by the editor first then I will proceed the reviewers.

**Response to editor**

1. Please provide additional details regarding participant consent. In the ethics statement in the Methods and online submission information, please ensure that you have specified (1) whether consent was informed and (2) what type you obtained (for instance, written or verbal, and if verbal, how it was documented and witnessed)

**Response:** I have adequately included these in the ethics statement in the methods section.

2. Please amend your detailed Financial Disclosure statement

**Response:** This is also clearly written in Financial Disclosure statement

3. In the online submission form, you indicated that "The datasets used and analyzed during the present study are available from the corresponding author on reasonable requests".

**Response:** I have included the English and Amharic version questionnaire used for my data collection within the manuscript. It is also uploaded as supplementary information. The questionnaire alone is sufficient for the replication of this study.

**Response to Additional Editor Comments**

1. Add some evidences of diabetes-related self-care practices of Ethiopia in Introduction

**Response:** I already added it the manuscript. You can look at the revised manuscript on the track change.

2. How many public health institutions were included in this study?

**Response:** A total of four public health institutions were included in the study. I included this in the revised manuscript.

3. The sampling fraction was: 815/392= 2.1. From where the authors used the number 292?

**Response:** I am not clear with the number 292. I can’t get the number 292 in the submitted manuscript. For more information regarding the sampling procedure; I have included the sampling procedure flow chart with figure.

4. Need more information about data collection. Authors mainly described about the questionnaire.

**Response:** I have added who collected the data, and how the data quality maintained in the revised manuscript.

5. Add a supplementary file of the questionnaire that used to collect data

**Response:** The questionnaire used for the data collection was added as a supplementary file.

6. Add a section of ‘Operational definition’ to define the key variables of interest

**Response:** Operational definition for the key variables was added in the revised manuscript.

7. Table 3: Mean score of self-care practices in North East Ethiopia, 2021. How mean score was calculated is not clear. Describe clearly in the method section.

**Response:** I already included this in the method section of the revised manuscript.

8. In the first paragraph of discussion, give a summary of objective-wise findings.

**Response:** I addressed this comment the revised manuscript.

9. Discussion need to be re-write. Add more evidences from global research, compare, and mention the causes of differences in the findings in between them.

**Response:** The discussion part was re-write. Evidences from global researches were included in the revised manuscript.

10. Need language and grammatical correction using professional

**Response:** I corrected the language and grammar with professional.

**Response to Reviewer #1**

Reviewer #1: Thanks to the author for their efforts, dedication and nice work. The topic of the study is very much essential to explore the present situation of self care practices of DM among people of LMICs like Ethiopia. I have found no major issues in the draft , though i will suggest the author to add an conceptual framework of the study. Along with that if they add another flow chart of their sample collection procedure, it will be eye catchy, but its not mandatory. Overall, this is a nice attempt.

**Response:** I would like to appreciate the reviewer for giving me a recognition for my hard work. After saying this, I would like to address points raised by the reviewers.

As per your comments, I already included conceptual framework, and sample collection procedure flow chart in the revised manuscript. Thank you!

**Response to Reviewer #2**

Thank you for the manuscript. I found major fundamental flaws in Introduction and Methodology section. The scope of self practice is not clear and is not supported by sufficient scientific literature. Methodology wise it is not scientifically sound and robust, and does not follow conceptual framework. Ethics is not clearly expressed. It needs a lot of effort to make it scientifically sound.

**Response:** I would like to appreciate the reviewer for giving a constructive comment. After saying this, I would like to address points raised by the reviewers.

I have included evidences of diabetes-related self-care practices of Ethiopia in Introduction section. The methods section also revised. The ethics part is also addressed. I have tried my best to revise the manuscript based on your comments. Thank you!
