# Supplementary material for: Self-care practice and its barriers among diabetes patients in North East Ethiopia: A facility-based cross-sectional study
Source: PLOS Glob Public Health. 2024 Feb 15;4(2):e0002036. doi: 10.1371/journal.pgph.0002036 (PMC10868755; doi:10.1371/journal.pgph.0002036)
Supplement: Attachment — Submitted filename: Manuscript (6)_Reviewed.docx [file pgph.0002036.s003.docx]

***Self-Care Practice and its Barriers among Diabetes Patients in North East Ethiopia: A facility-based cross-sectional Study***

Adisu Asefa, ^1^ Abebe Muche Belete, ^1^ Feredegn Talarge, ^1^ Daniel Molla^1^

^1^Department of Biomedical Science, College of Medicine, Debre Berhan University, Debre Berhan, Ethiopia

* Corresponding author: Adisu Asefa P.O.Box: 445, Phone: Tel +251912464355,

Email [sadamasefadb@gmail.com](mailto:sadamasefadb@gmail.com)

**Abstract**

**Background**: Diabetes prevention and management through self-care practice is critical to reducing severe complications and death due to diabetes. Data on the prevalence of self-care practices will help us to design and implement prevention and management strategies to foster adherence and compliance with the interventions. This study was intended to assess self-care practice and its barriers among diabetes patients in Northeast Ethiopia. A facility-based cross-sectional study was conducted among diabetes patients visiting Debre Berhan Town Public Health Institutions from March 10, 2021- April 10, 2021. A systematic random sampling technique was utilized to select 392 samples. Data were collected using a structured questionnaire adapted from Summary of Diabetes Self-Care Activities Measures. Reliability analysis was done using Cronbach's alpha test, and the Hosmer and Lemeshow test also checked for model fitness. Bivariate and multivariable binary logistic regression was done to identify the factors associated with dietary practices. For all statistically significant tests, p- a value < 0.05 was used as a cut-off point.

**Result:** The mean age of the respondents was 47.1 years, with a standard deviation (SD) of ± 13.4 years. The mean adherence to self-care practice was 29.00 ± 10.37 SD. More than half, 218 (61.1%) of the study subjects had poor self-care practice. In the multivariable logistic regression analysis, being a governmental worker and having social support from partners, family, and friends showed a statistically significant association with good self-care practice.

**Conclusion:** The current study showed that the overall level of self-care practice of study subjects was poor. Therefore, health facilities should provide adequate health education and promotion activities to enhance patients’ level of adherence. In addition, families, partners, or friends of diabetes patients should be informed about their essential roles in patients' self-care practice.

**Keywords:** type-2 diabetes mellitus, dietary, self-care practice

**Introduction**

In most developed countries, diabetes is the fourth or fifth leading cause of death, and increasing evidence shows that it has reached epidemic proportions in many developing countries [1, 2].

Type 2 diabetes results from the interaction between genetic capacity, high-risk behaviours, and environmental risk factors [3]. According to the 2019 International Diabetes Federation (IDF) report, approximately 463 million adults (20-79 years) have diabetes; by 2045, this number will increase to 700 million [4].

Ethiopia is one of the sub-Saharan countries, and the double burden of its population is increasing epidemiological risks; namely, cancer, cardiovascular diseases, chronic respiratory diseases, and diabetes, which cause 60% of global deaths, but receive just 3% of international development assistance for health [5].

The foundation of diabetes management is adherence to lifestyle interventions, including healthy eating, regular physical exercise, smoking cessation, and maintaining a healthy weight [6]. Diabetes-related complications and mortality are prevented and controlled primarily by adopting healthy lifestyle habits. However, this is a challenging problem in developing countries where the quality of healthcare services could be better [7].

Regardless of the type of diabetes, 95% of diabetes control is based on diet and lifestyle interventions, and the patient or his family usually provides 95% of self-care. Therefore, diabetic patients must adjust their behaviour, such as changing their lifestyles to change their diet and physical activity level and following prescribed treatments to prevent diabetes complications, which can be fatal, especially for people [8-12].

A large percentage of chronic illnesses are prevented and managed through the reduction of the four main shared behavioural risk factors: tobacco use, physical inactivity, the harmful use of alcohol, and unhealthy diets (referred to as modifiable risk factors). For example, up to 80% of heart disease, stroke, and type 2 diabetes and about 40% of cancers could be prevented by controlling these risk factors [5].

Evidence from a systematic review and meta-analysis study conducted in Ethiopia showed that the pooled prevalence of good diabetes self-care practice among diabetic patients was 49%. This study characterized the effective regimen of diabetes self-care based on pooled prevalence. The pooled estimate for diet was 50%, self-blood glucose monitoring was 28%, recommended physical activity was 49%, and diabetic foot care was 58% [13].

A study conducted in Northern Ethiopia revealed that the overall good self-care practice was 50.3%. This study also showed that 39.3% and 54.9% of the study participants had good exercise, and diet adherence, respectively [14]. Another study from Ethiopian General Hospital indicated that (63.1%) of study participants had good self-care practices. In addition, this study identified that patients who had a glucometer at home had social support and received a diabetes education was statistically associated with good self-care practice [15].

According to a study carried out in Addis Ababa, Ethiopia, three fourth (75.9%) of study patients did not follow the recommended diet management, (and 83.5%) did not adhere to self-monitoring of blood glucose levels. In comparison, a few (4.3%) respondents did not take the administered diabetes medications [16].

However, more about self-care practice in North-East Ethiopia needs to be studied. To prevent complications related to diabetes, it is essential to find out how patients with type 2 diabetes adhere to self-care measures. Self-care practice is vital for health sector stakeholders to design and implement prevention strategies for diabetes-related complications; it also reduces medicine's direct and indirect costs. Therefore, the current study might help identify gaps in areas of self-care practice which are essential in many aspects, both for the individual’s well-being and from a socioeconomic perspective (S1 Fig).

**Materials and Methods**

**Study design and setting**

A facility-based cross-sectional study was carried out among diabetes patients attending Debre Berhan Town Public Health Institutions, North-East Ethiopia, from March 10, 2021- April 10, 2021.

**Sample Size Determination**

According to a previous study conducted in Ethiopian teaching hospitals, adherence to dietary recommendations was 44.3% [17]. Therefore, the minimum sample size required for this study was determined by using the single population proportion formula.

(ni = (Zα/2)2 p (1-p) /d2), Taking p = 44.3%, a 5% precision (d) level with a 95% confidence interval plus a 10% non-response rate was added. A population correction formula was also utilized. The final sample size was = 392.

**Sampling Procedures and Techniques**

Four public health institutions in Debre Berhan town, North- East Ethiopia, were included in the study. Based on the count from the registration books on diabetic follow-up clinics, the average number of diabetes attending the selected institutions per month was 815.

A systematic random sampling technique was used among 815 patients coming to these institutions for a follow-up service. The sampling fraction was: 815/392= 2.1. The first sample was selected by using a simple random sampling technique. Then, every two patients from each public health institution were included in the study until the calculated sample size was achieved (S2 Fig).

**Study Variables**

**Dependent variable**: Self-care practice

**Independent Variables**:

**Sociodemographic variables**: age, sex, marital status, educational status, occupation, and income

**Patient health status variables**: duration of the disease, family history of diabetes, treatment intensity, diabetes complications, social support, cigarette smoking status.

**Data Collection Tools and Procedures**

Data were collected using a structured questionnaire adapted from the Summary of Diabetes Self-Care Activities Measure (SDSAM) [18]. The questionnaires consist of four parts: sociodemographic characteristics, patient health status, a summary of diabetes self-care activities measure, and barriers to lifestyle and dietary pattern interventions. Summary of Diabetes Self-Care Activities Measure (SDSAM) asked about the diabetes self-care activities during the past seven days. The Summary of Diabetes Self-Care Activities Measure (SDSAM) included five components: diet, exercise, blood sugar testing, foot care, and smoking. Scores were calculated for each of the five regimen areas assessed by the SDSCA: Diet, Exercise, Blood-Glucose Testing, Foot Care, and Smoking Status. The mean value of lifestyle and dietary practices for the study participants was calculated using a score ranging from 0 to 7 for each item of the five SDSCA domains. For general diet = the mean number of days for items 1 and 2, specific diet = a mean number of days for items 3 and 4, reversing item 4 (0=7, 1=6, 2=5, 3=4, 4=3, 5=2, 6=1, 7=0), exercise = a mean number of days for items 5 and 6, blood-glucose testing = a mean number of days for items 7 and 8, foot-Care = a mean number of days for items 9 and 10, smoking Status = Item 11 (0 = non-smoker, 1 = smoker), and several cigarettes smoked per day [18].

The reliability of the SDSCA questionnaire was tested using Cronbach's alpha test. The overall reliability was 0.757. The questionnaire was prepared in English, translated into the Amharic version, and then back to the English version. Five BSc health professionals were recruited to collect the data, and two BSc/ MSc health professionals supervised the data collection process. The training was given to the data collectors and supervisors for three days to maintain data quality. Timely supervision was carried out by the principal investigator daily.

**Data Processing and Analysis**

The data was entered into EPI data manager version 3.3 and analyzed using IBM SPSS Statistics version 22. Reliability was analyzed using Cronbach’s alpha test. Model fitness was also checked by Hosmer and Lemeshow test. A summary of descriptive statistics was computed for most variables. Bivariate analysis was done to determine the presence of an association between lifestyle and dietary pattern intervention practice with other variables. To avoid unstable estimates in the subsequent model, only variables that reached a p-value less than 0.2 at bivariate analysis were included in the subsequent model analysis. Multiple logistic regression analysis was applied to describe the functional independent barriers of adherence to self-care practice. A point estimate of Odds ratio (OR) with a 95% confidence interval (CI) was determined to assess the strength of association between independent and dependent variables. For all statistically significant tests, p- a value <0.05 was used as a cut-off point.

**Operational Definition**

**Self-care practice**: Refers to self-care activities such as following a diet plan, avoiding high-fat foods, regular physical activity, self-glucose monitoring, and administration of medication engaged for the past seven days, which is measured by Summary of Diabetes Self-Care Activities [18].

**Poor self-care practice**: Those patients' adherence to self-care behaviours scored below the mean of self-care behaviour items.

**Good self-care practice**: Those patients' adherence to self-care behaviours scored above the mean of self-care behaviour items.

**Family history of diabetes**: having a history of diabetes of their parents and first- and second-level relatives as self-reported.

**Lifestyle management:** non-pharmacological management such as physical exercise, foot care activities, and smoking cessation is designed to treat the problems of type 2 DM patients.

**Self-care behaviours mean score**: (Number of days patient practiced specific behaviour)/ (Total number of days under questions designed for that behaviour).

**Results**

A total of 357 study participants were successfully involved in the study yielding a response rate of 91.1%. The majority (61.1%), (71.7%), and (81%) of study participants were men, married, and able to read and write, respectively. Their ages ranged from 18 to 86 years, with a mean age of 47.1 ± 13.4 years. The average monthly income for the study participants was 4070 ETB. Most of the participants, 95 (26.6%), were working private jobs (Table-1).

Table 1: Sociodemographic characteristics of study participants, North East Ethiopia, 2021.

| **Sociodemographic Variables Frequency (%)** | |  |
| --- | --- | --- |
| **Sex**  Male  Female | 218 (61.1)  139 (38.9) | |
| **Age group**  15-24  25-64  > 64 | 14 (3.9)  306 (85.7)  37 (10.4) | |
| **Marital status**  Single  Married  Widowed  Divorced | 53 (14.9)  256 (71.7)  24 (6.7)  24 (6.7) | |
| **Occupation**  Physical labourer  Teacher  Health professional  Business worker  Engineer  Private worker  Housewife  Driver  Others | 12 (3.4)  31 (8.7)  18 (5)  60 (16.8)  6 (1.7)  95 (26.6)  67 (18.8)  13 (3.6)  55 (15.4) | |
| **Educational status**  Unable to read and write.  Able to read and write.  Primary school  Secondary school  College or University | 68 (19)  99 (27.7)  46 (12.9)  69 (19.3)  75 (21) | |

**Health Status Characteristics of Participants**

The study result revealed that most 316 (88.5%) respondents had no family history of diabetes. Most (95.2%) of the respondents were non-smokers. Approximately three fourth of the respondents (72%) have incurred diseases more significant than five years of duration. Of the total respondents, 296 (82.9%) of them had no diabetes-related complications. More than half (58.5%) of them had no social support (partner, family, friend, etc.) (Table 2).

Table 2: Health status characteristics of diabetes, North East Ethiopia, 2021.

**Variables	Frequency (%)**

**Duration of diseases**

Less than one year	36 (10.1)

From one to five years	64 (17.9)

Greater than five years	257 (72)

**Family history of diabetes**

Yes	41 (11.5)

No	316 (88.5)

**Treatment intensity**

Oral hypoglycemic agent	283 (79.3)

Insulin therapy	45 (12.6)

Combination	29 (8.1)

**Social support**

Yes	148 (41.5)

No	209 (58.5)

**Currently having glucometer**

Yes	32 (9)

No	325 (91)

**Diabetes complication**

Yes	61 (17.1)

No	296 (82.9)

**Cigarette smoking**

Yes	17 (4.8)

No	340 (95.2)

**Self-care practices adherence**

The frequency of respondents in a diabetes self-care behaviour defined by the number of days per week was analyzed by calculating the mean score, which refers to the average number of days respondents adhered to self-care measures in the previous seven days. Based on this, the overall mean and standard deviation of adherence to self-care practice were 29.00 ± 10.37 (Table 3).

Table 3: Mean score of self-care practices in North East Ethiopia, 2021.

**Self-care measures (0-7 days)	Mean (SD)**

**Overall self-care adherence**	**29.00 ± 10.37**

Dietary pattern	16.47 **± 5.08**

General diet	**6.35 ± 3.66**

Specific diet	**10.12 ± 1.98**

Physical exercise	**4.78 ± 2.56**

Blood glucose	**3.39 ± 2.59**

Foot care	**4.35 ± 3.61**

The overall level of adherence to self-care practice was categorized as good and poor adherence using an average mean score. The current study revealed that 218 (61.1%) of the study subject had poor self-care practices. Two hundred six (57.7%) of the study subjects had poor dietary pattern adherence, while two hundred one (56.3%) had poor exercise adherence.

**Self-care recommendations for lifestyle and dietary pattern intervention practice**

More than half (58.3%) of the study subjects were advised about eating low dietary fat in their meals. Similarly, 184 (51.5%) were advised about eating at least five servings of fruits and vegetables daily. However, 66 (18.5%) had yet to give any advice about their dietary pattern.

Three hundred two (84.6%) and 97 (27.2) of the respondents were advised regarding adherence to low-level physical exercise (such as walking) daily and the importance of participating in sports activities (specific amount, type, duration, and level of exercise), respectively. But 48 (13.4%) reported that they had not given any advice about physical exercise.

The health care team (doctor, nurse, and health officer) has also advised regarding testing blood sugar levels using a drop of blood from a finger prick using a colour chart. Two hundred ninety-four (82.4%) reported that they were advised about the importance of testing blood sugar levels, but 54 (15.1%) reported that they had not given any advice about blood sugar testing.

Two hundred seventy-one (75.9%) of them were advised on how to take diabetes pills to control blood sugar levels.

**Barriers to self-care practices**

In order of magnitude, the stated barriers for poor dietary adherence included, (62.3%), (61.6%), (40.6%), (21.6%), and (15.7%) of the study subjects described poor self-discipline, difficult situations at home, financial constraint, lack of information about the list of healthy diets, eating out in a restaurant, and eating at the neighbourhood, family or other respectively.

In order of magnitude, the stated barriers to physical exercise adherence included, (79.6%), (30.5%), (and 11.5%) of the study subjects described very cold weather conditions of the town, lack of information about the benefit of exercise and how it should be done, and the belief that physical exercise exacerbates the conditions of the disease respectively (Table 4).

Table 4: Barriers to lifestyle and dietary intervention among diabetes in North East Ethiopia, 2021.

**Barriers to dietary pattern and exercise adherence	Frequency (%)**

**Yes (%)	No (%)**

**Dietary pattern**

Eating out (in a restaurant)	63 (17.6%)	294 (82.4%)

Financial constraints	145 (40.6%)	212 (59.4%)

Self-discipline	222 (62.3%)	135 (37.8%)

Eating at another's home	56 (15.7%)	301 (84.3%)

Difficult situation at home	220 (61.6%)	137 (38.4%)

Lack of information on a healthy diet	77 (21.6%)	280 (78.4%)

**Physical exercise**

The very cold weather condition of the town	284 (79.6%)	73 (20.4%)

Lack of exercise partner	49 (13.7%)	308 (86.35)

Lack of specific location	107 (30%)	250 (70%)

Criticism	26 (7.3%)	331 (92.7%)

Lack of information about the benefit of exercise	109 (30.5%)	248 (69.5%)

The belief that exercise exacerbated diabetes mellitus	41 (11.5%)	316 (88.5%)

Others	8 (2.2%)	349 (97.8%)

**Factors associated with self-care practice**

During the multivariable logistic regression analysis, government workers were 7.06 times more likely to have good self-care practices than other professions (AOR= 7.06; 95% CI: (1.61-30.9)). Concerning educational status, those study subjects who were unable to read and write, able to read and write, and attending primary school were 72.8%, 60%, 66%, respectively, less likely to have good self-care practice than those study subjects who attended college or university (AOR=0.28; 95% CI: (0.09-0.83), AOR= 0.40; 95% CI (0.16-0.99), AOR= 0.34; 95% CI(0.12-0.98)), respectively.

Study subjects with social support from partners, family, and friends were 7.40 times more likely to have good self-care practices than those without family support (AOR=7.40 (4.03-13.57)). Furthermore, study subjects who had a self-glucometer machine in their home were 5.82 times more likely to have good self-care practice than those who had no self-glucometer machine in their home (AOR= 5.82(1.83-18.4)) (see table 5)

During the multivariable logistic regression analysis, those who were 15-24 years age group were 93% less likely to adhere to good dietary practices than those who were > 64 years age group. Furthermore, those with social support from partners, family, and friends were 5.83 times more likely to adhere to good dietary practices than those without family support (AOR=5.83(3.01-11.3)). Finally, study subjects eating out in restaurants were 76% less likely to adhere to good dietary practices than those not eating in restaurants.

Male study subjects were 2.01 times more likely to adhere to good physical exercise practice than females (AOR=2.01 (1.02-3.98)).

Table-5: Factors associated with self-care practice among diabetes in North East Ethiopia, 2021.

| **Variables COR (CI) AOR (CI) P-value** | | | |
| --- | --- | --- | --- |
| ***Sociodemographic***  **Age group**  15-24  25-64  >64  **Marital status**  Single  Married  Widowed  Divorced  **Occupation**  Private worker  Governmental worker  Housewife  **Educational status**  Unable to read and write  Able to read and write  Primary school  Secondary school  College or university  **Income category**  Lower-income  Higher-income  ***Health status characteristics***  **Duration of diseases**  Less than one year  From one to five year  Greater than five year  **Treatment intensity**  Oral hypoglycemic agent  Insulin therapy  Combination  **Social support**  Yes  No  **Currently glucometer**  Yes  No | 2.06(0.48-8.82)  3.76(1.52-9.29)  1  3.36(1.15-9.79)  2.02(0.77-5.25)  0.27(0.04-1.51)  1  3.69 (1.70-7.98)  17.6 (6.62-47.0)  1.35 (0.53-3.40)  1  0.07(0.03-0.17)  0.14(0.07-0.28)  0.12(0.05-0.29)  0.31(0.15-0.63)  1  0.18(0.11-0.29)  1  2.20(1.08-4.46)  1.20(0.68-2.11)  1  0.52(0.24-1.13)  0.29(0.11-0.79)  1  7.56(4.68-12.20)  1  10.27(3.84-27.39)  1 | 0.27(0.03-1.97)  0.89(0.25-3.21)  1  3.10(0.76-12.60)  1.83(0.59-5.65)  0.50(0.07-3.46)  1  1.92 (0.60-6.06)  7.06 (1.61-30.9)  1.48 (0.44-4.98)  1  0.28(0.09-0.83)  0.40(0.16-0.99)  0.34(0.12-0.98)  0.66(0.27-1.65)  1  0.68(0.31-1.48)  1  1.77(0.63-4.99)  1.60(0.73-3.47)  1  3.29(1.10-9.77)  1.17(0.32-4.29)  1  7.40(4.03-13.57)  1  5.82(1.83-18.4)  1 | 0.19  0.86  0.11  0.29  0.48  0.26  **0.01***  0.52  **0.02***  **0.04***  **0.04***  0.38  0.33  0.27  0.23  **0.03***  0.80  **0.01***  **0.01*** |

****Statistically significant association***

**Discussion**

The majority of diabetic patients in our study lacked comprehensive self-care practice. Most of the study participants undermine the significance of the elements of self-care practice either due to lack of information, lack of resources/poverty, lack of social support or negligence.

The study showed that the overall mean and standard deviation of adherence to diabetes self-care practice measures was 29.00 ± 10.37. The current finding was lower than other West Shoa Zone studies, which were 39.8 ± 9.5 [24]. This difference might be due to differences in socioeconomic status. However, this finding was higher than a study conducted in public hospitals found in western Ethiopia, which was 23.09 ±6.55 [25]. Therefore, the high mean score for self-care practice indicates good adherence, which might significantly contribute to disease control. Furthermore, it reduces complications related to diseases.

The current study revealed 61.1% of the subjects had poor self-care practices. Comparable findings were noted in a study conducted in Jeddah; Saudi Arabia showed that 62% of participants had inadequate practice in self-management [33]. Similar findings were revealed in a study conducted at public hospitals in western Ethiopia, a study done at Jimma University Specialized Teaching Hospital and a study from Harari, of which 57.3%, 55%, and 60.7% of the participants had poor self-care practice, respectively [26, 27, 28 ]. However, this finding was lower than a multinational web-based survey conducted in the UK, US, Australia, and Japan. The level of self-care practice was highest in Japan (54.9%), followed by the UK (43.1%), the USA (42.5%), and Australia (40.4%) [34]. The desire for self-reliance could explain this difference in participants from all four countries. The second common reason could be the cost-effectiveness of practicing self-care measures in these countries [34]. Similarly, the current finding was higher than the result of Chali et al., which was 45.7% [29] and the finding from the systematic review and meta-analysis conducted by Dagnew et al., which was 48.88% (32). This difference might be due to the scope of the study, socioeconomic differences, and sample size variations.

This study also showed that study subjects who were unable to read and write, able to read and write, and attending primary school were 72.8%, 60%, and 66%, respectively, less likely to have good self-care practice than those respondents who had college or university degrees. Studies from other settings showed that diabetes patients with a higher level of educational status are engaged more in self-care practice [30, 31].

The current study revealed that (57.7%) of the study subjects had poor dietary pattern practice. Comparable findings were noted in a study conducted in Addis Ababa which indicated 51.4% was a poor level of adherence [19].

The current finding revealed that social support group was one predictor factor that affects dietary pattern practice in diabetes patients; in this study, respondents who had social support from partners, family, and friends were 5.83 times more likely to adhere to good dietary practice than those who had lack of family support (AOR=5.83(3.01-11.3)). This finding was supported by the study conducted in Benishangul Gumuz [21] and Jimma [22]. Social support from partners, family members, and friends positively predicts diet and exercise recommendations adherence. "Family and friends play both supportive and obstructive roles. Participants identified both positive and negative contributions of family and community members to their diabetes management [20]. Study subjects who were eating out in restaurants were 76% less likely to adhere to good dietary practices. These findings are comparable with a study conducted by Ayele et al. [23].

**Conclusion**

The current study showed that the overall level of self-care practice was poor. Based on our findings, we recommend that health professionals working in Debre Berhan town public health institutions should provide adequate health education and promotion activities to enhance patient’s level of adherence to self-care activities measures, and families, partners, or friends of diabetes patients should be informed about their essential roles in encouraging, reinforcing and supporting the patients for the better adherence of self-care activities measure.

**Ethics Statement**

This study was conducted after ethical approval letters obtained from the Institute of research ethics and the review board (IRB) of the College of Health Science, Debre Berhan University, Ethiopia, complied with the Declaration of Helsinki. First, the Institute of Research and ethics board committee (IRB) reviewed and looked at the study's originality, feasibility, and ethical aspects. Following thorough discussion, the committee approved the research proposal by authors with ethical approval using reference number SPH/112/2020. Then permission was taken from the hospital and health centers' higher management, and data were collected after obtaining oral of written informed consent from the study participants. To keep confidentiality, codes were used, and unauthorized persons did not have access to the data.

**List of Abbreviations**

IDF, International Diabetes Federation; SDSAM, Summary of Diabetes Self-Care Activities Measure

**Acknowledgments**

We want to thank Debre Berhan Referral Hospital, Kebele 04, 07, and 08 health facilities for their kind cooperation and support. We also acknowledge Debre Berhan University for its kind cooperation in conducting the study.

**Declarations**

Financial Disclosure Statement

The authors of this manuscript fully funded this study.

**Data Sharing Statement**

The datasets used and analyzed during the present study are available from the corresponding author on reasonable requests.

**Disclosure**

The authors declare that they have no competing interests in this work.

**References**

1. Steyn NP, Mann J, Bennett PH, Temple N, Zimmet P, Tuomilehto J, et al. Diet, nutrition and the prevention of type 2 diabetes. Public Health Nutr. 2004; 7(1A):147–65. [PubMed]
2. Amos AF, McCarty DJ, Zimmet P. The rising global burden of diabetes and its complications: estimates and projections to the year 2010. Diabet Med. 1997; 14 Suppl 5:S1–85. [PubMed]
3. Tuomilehto J, Lindstrom J, Eriksson JG, Valle TT, Hamalainen H, Ilanne-Parikka P, et al. Prevention of type 2 diabetes mellitus by changes in lifestyle among subjects with impaired glucose tolerance. N Engl J Med. 2001; 344(18):1343–50. Doi: 10.1056/NEJM200105033441801. [PubMed] [CrossRef]
4. International Diabetes Federation. 9th edition IDF Diabetes Atlas. Brussels: (2019). Retrieved from: <http://www.diabetesatlas.org>
5. EPHA report. The new EPHA booklet provides the necessary information on the existing situation of Non-Communicable diseases in Ethiopia. 2012.
6. K. G. Alberti, P. Zimmet, and J. Shaw, “International Diabetes Federation: a consensus on type 2 diabetes prevention, “Diabetic Medicine, vol. 24, no. 5, pp. 451–463, 2007.
7. World Health Organization, WHO Country Cooperation Strategy at a Glance, World Health Organization, Geneva, 2009.
8. Zhou Y, Liao L, Sun M, He G. Self-care practices of Chinese individuals with diabetes. Exp Ther Med. 2013; 5: 1137-1142.
9. Shrivastava SR, Shrivastava PS, Ramasamy J. Role of self-care in management of diabetes mellitus. J Diabetes Metab Disord. 2013; 12: 14.
10. Aanand V. Implementation plans and self-monitoring of blood glucose in diabetics. [Dissertation], Michigan University. 2009; 2: 60.
11. Okolie VEI, Ihanacho O. Knowledge of diabetes management and control by diabetic patients at Federal Medical Center UmuahiaAbia State Nigeria. International Journal of Medicine and Medical Sciences 1. 2009.
12. Maizlish NA, Shaw B, Hendry K. Glycemic control in diabetic patients served by community health centers. Am J Med Qual 2004; 19: 172-179.
13. Ketema, D.B., Leshargie, C.T., Kibret, G.D. et al. Level of self-care practice among diabetic patients in Ethiopia: a systematic review and meta-analysis. BMC Public Health 20, 309 (2020). https://doi.org/10.1186/s12889-020-8425-2.
14. Zewdie S, Moges G, Andargie A, Habte BM. Self-Care Practice and Associated Factors Among Patients with Type 2 Diabetes Mellitus at a Referral Hospital in Northern Ethiopia–a Mixed Methods Study
15. Tiruneh, S.A., Ayele, A.A., Emiru, Y.K. et al. Factors influencing diabetes self-care practice among type 2 diabetes patients attending diabetic care follow-up at an Ethiopian General Hospital, 2018. J Diabetes Metab Disord 18, 199–206 (2019). <https://doi.org/10.1007/s40200-019-00408-z>
16. Bonger Z, Shiferaw S, Tariku EZ. Adherence to diabetic self-care practices and its associated factors among patients with type 2 diabetes in Addis Ababa, Ethiopia. Patient preference and adherence. 2018;12:963
17. Mohammed et al. Adherence to the dietary recommendation and associated factors among diabetic patients in Ethiopia teaching hospitals. Pan African Medical Journal. 2019; 33:620. Doi 10.11604/pamj.2019.33.260.14463.
18. Deborah J, Sarah E, & Russell E. Summary of Diabetes Self-Care Activities Measure; Diabetes Care. 2000; 23:943–950.
19. Amelmal W, Solomon M, & Mekonnen A. Dietary practice and associated factors among type-2 diabetic patients in Addis Ababa. Springer Plus; 2015:4:15.
20. A. Ayele, Y. K. Emiru, S. A. Tiruneh, B. A. Ayele, A. D. Gebremariam, and H. G. Tegegn, “Level of adherence to dietary recommendations and barriers among type 2 diabetic patients: a cross-sectional study in an Ethiopian hospital,” Clinical Diabetes and Endocrinology. 2018; vol. 4, no. 1, pp. 120– 126,
21. Chali SW, Salih MH, Abate AT. Self-care practice and associated factors among diabetes mellitus patients on follow up in Benishangul Gumuz regional state public hospitals, Western Ethiopia: a cross-sectional study. BMC research notes. 2018 Dec; 11(1):1-8.
22. Hailu E, Mariam WH, Belachew T, Birhanu Z, Health F, Birhanu Z, Hailu E, Mariam WH, Belachew T, Birhanu Z, Health F, Birhanu Z, et al. Self-care practice and glycemic control amongst adults with diabetes at the Jimma University Specialized Hospital in southwest Ethiopia. Afr J Prim Health Care Fam Med. 2012; 4(1):1–6. [https://doi.org/10.4102/phcfm .v4i1.311](https://doi.org/10.4102/phcfm%20.v4i1.311)
23. Ayele A, Emiru K, Tiruneh A, Ayele A, Gebremariam D & Tegegn G. Level of adherence to dietary recommendations and barriers among type 2 diabetic patients: A cross-sectional study in an Ethiopian hospital. Clinical Diabetes and Endocrinology; 2018. 4(21), 120-126. DOI: 10.1186/s40842-018-0070-7
24. Guru Y, Gela D, Aga F. Factors associated with self-care practice among adult diabetes patients in West Shoa Zone, Oromia Regional State, Ethiopia. BMC health services research. 2018 Dec; 18(1):1-8.
25. Adugna O, Getu M, Muktar A Reta T, Ashenafi H, & Eba A. Predictors of Adherence to Self-Care Behavior among Patients with Diabetes at Public Hospitals in West Ethiopia. Diabetes, Metabolic Syndrome and Obesity: Targets and Therapy 2020:13 3277–3288
26. Olema A, Mosisa G, Abadiga M, Tsegaye R, Habte A, Abdissa E. Predictors of Adherence to Self-Care Behavior Among Patients with Diabetes at Public Hospitals in West Ethiopia. Diabetes, Metabolic Syndrome and Obesity: Targets and Therapy. 2020; 13: 3277.
27. Yemane T, Belachew T, Asaminew BBO. Type 2 diabetes mellitus in Jimma Town SouthwestEthiopia. Ethiopia J Health Sci. 2007; 17 (2):107
28. Ayele K, Tesfa B. Self-care behaviour among patients with diabetes in Harari, Eastern Ethiopia. Health Belief Model Perspect. 2012; 7(4)
29. Chali SW, Salih MH, Abate AT. Self-care practice and associated factors among diabetes mellitus patients on follow up in Benishangul Gumuz regional state public hospitals, Western Ethiopia: a cross-sectional study. BMC research notes. 2018 Dec; 11(1):1-8.
30. Bai Y-L, Chiou CP, Chang Y-Y. Self-care behaviour and related factors in older people with type 2 diabetes. J Clin Nurs. 2009; 18:3308–15.
31. Albikawi ZF, Abuadas M. Diabetes self-care management behaviours among Jordanian type Two diabetes patients. Am Int J Contemp Res. 2015; 5(3):87–95.
32. Dagnew B, Debalkie Demissie G, Abebaw Angaw D. Systematic review and meta-analysis of good self-care practice among people living with type 2 diabetes mellitus in Ethiopia: a national call to bolster lifestyle changes. Evidence-Based Complementary and Alternative Medicine. 2021 February 22February 22; 2021.
33. AlQahtani A H, Alzahrani A S, Alzahrani S H, et al. Levels of Practice and Determinants of Diabetes Self-Care in Primary Health Care in Jeddah City, Saudi Arabia. Cureus. June 25June 25, 2020; 12(6): e8816. doi:10.7759/cureus.8816
34. Mun S, Park JH, Baek SM, Lee M, Choi SM, Lee S. Self-care use patterns in the UK, US, Australia, and Japan: a multinational web-based survey. Integr Med Res. 2016 Jun;5(2):151-160. Doi 10.1016/j.imr.2016.03.001. Epub 2016 March 9 March 9. PMID: 28462110; PMCID: PMC5381426.
35. N. Zeng B, Sun W, Rebecca A, Li C, Liu T. Towards a conceptual model of diabetes self-management among Chinese immigrants in the United States. Int.J. Environ.Res. Public Health 2014, 11, 6727-6742; doi: 10.3390/ijerph110706727

**Supporting information**

**S1 Fig. Conceptual framework for studying barriers to self-care practices among diabetes** patients.

**S2 Fig. Flow chart indicating sampling procedure in Debre Berhan Town Public Health Institutions**

**S1Table. Sociodemographic characteristics of study participants, North East Ethiopia, 2021.**

**S2Table. Health status characteristics of diabetes, North East Ethiopia, 2021.**

**S3Table. Mean score of self-care practices in North East Ethiopia, 2021.**

**S4Table. Barriers to lifestyle and dietary intervention among diabetes in North East Ethiopia, 2021.**

**S5Table. Factors associated with self-care practice among diabetes in North East Ethiopia, 2021.**

**S1Annex. Questionnaire**
