# Supplementary material for: Self-care practice and its barriers among diabetes patients in North East Ethiopia: A facility-based cross-sectional study
Source: PLOS Glob Public Health. 2024 Feb 15;4(2):e0002036. doi: 10.1371/journal.pgph.0002036 (PMC10868755; doi:10.1371/journal.pgph.0002036)
Supplement: Attachment — Submitted filename: Response to Reviewers 2.docx [file pgph.0002036.s004.docx]

Date: 22 Nov 2023

**Dear the editor and reviewers of PLOS Global Public Health,**

I would like to appreciate all of you for considering my manuscript for your journal. I highly appreciate your patience to give me adequate time to revise the manuscript based on the points raised by the editor and reviewers.

I would like to thank the editor and reviewers for giving me a nice comments and suggestions. I appreciate the points raised during the review process. After saying this, I would like to address the points raised by the editor first then I will proceed the reviewers.

**Response to editor**

1. Please provide a copy editing

**Response:** As per the editor suggestion, I have prepared a copy-editing document. This document will be attached in the submission of revised version of the manuscript.

2. Please provide additional details regarding participant consent. In the ethics statement in the Methods and online submission information, please ensure that you have specified (1) whether consent was informed and (2) what type you obtained (for instance, written or verbal, and if verbal, how it was documented and witnessed)

**Response:** I have adequately addressed these queries in the ethics statement in the methods section and online submission information.

**Response to Reviewer #1**

**Response:** I would like to appreciate the reviewer for giving me a recognition for my hard work.

As per your comments on the attachment, I have already corrected and responded to comments raised on the track change of the revised manuscript.

Thank you!
